# 

**Published:** 1895-11-23

**Authors:** 


					-ia*r*MwT2TLu ABSOLUTELY Pure, therefore BEST. Novkmbkb 23bd, 1895.
CADBURYS ^ ww w CADBURY'S
COCOA
"ZSUbl?^of^Ust purity at prescnt ^ ^ NO ALKALIES USED.
COCOA
'8 trieH HOSPl.TAL
No. 478. Vol. XIX.
Saturday,
Nov. 23rd, 1895.
WITH EXTRA NURSING SUPPLEMENT. mice twopence.
__ . . . . .. _ . _ , _ _ .. . Per Annum, 10s. 6d? post free.
[Registered at the General Post Office as a Newspaper for Monthly Parts, 6d.; post free, 8d.
transmission in the United Kingdom and Abroad.] Ditto per Annum, 8s. 6d., post free.

				

